# Iterative reconstruction of three-dimensional models of human chromosomes from chromosomal contact data

**DOI:** 10.1186/s12859-015-0772-0

**Published:** 2015-10-23

**Authors:** Jackson Nowotny, Sharif Ahmed, Lingfei Xu, Oluwatosin Oluwadare, Hannah Chen, Noelan Hensley, Tuan Trieu, Renzhi Cao, Jianlin Cheng

**Affiliations:** Department of Computer Science, Informatics Institute, University of Missouri, Columbia, MO 65211 USA

## Abstract

**Background:**

The entire collection of genetic information resides within the chromosomes, which themselves reside within almost every cell nucleus of eukaryotic organisms. Each individual chromosome is found to have its own preferred three-dimensional (3D) structure independent of the other chromosomes. The structure of each chromosome plays vital roles in controlling certain genome operations, including gene interaction and gene regulation. As a result, knowing the structure of chromosomes assists in the understanding of how the genome functions. Fortunately, the 3D structure of chromosomes proves possible to construct through computational methods via contact data recorded from the chromosome. We developed a unique computational approach based on optimization procedures known as adaptation, simulated annealing, and genetic algorithm to construct 3D models of human chromosomes, using chromosomal contact data.

**Results:**

Our models were evaluated using a percentage-based scoring function. Analysis of the scores of the final 3D models demonstrated their effective construction from our computational approach. Specifically, the models resulting from our approach yielded an average score of 80.41 %, with a high of 91 %, across models for all chromosomes of a normal human B-cell. Comparisons made with other methods affirmed the effectiveness of our strategy. Particularly, juxtaposition with models generated through the publicly available method Markov chain Monte Carlo 5C (MCMC5C) illustrated the outperformance of our approach, as seen through a higher average score for all chromosomes. Our methodology was further validated using two consistency checking techniques known as convergence testing and robustness checking, which both proved successful.

**Conclusions:**

The pursuit of constructing accurate 3D chromosomal structures is fueled by the benefits revealed by the findings as well as any possible future areas of study that arise. This motivation has led to the development of our computational methodology. The implementation of our approach proved effective in constructing 3D chromosome models and proved consistent with, and more effective than, some other methods thereby achieving our goal of creating a tool to help advance certain research efforts. The source code, test data, test results, and documentation of our method, Gen3D, are available at our sourceforge site at: http://sourceforge.net/projects/gen3d/.

## Background

The genetic information of all living organisms, known as deoxyribonucleic acid (DNA), gives the instructions for development and functioning of the organism and is, therefore, crucial for the organism to function. In humans, the DNA is organized into 46 chromosomes in the form of 23 pairs, each containing different parts of the entire DNA. The collection of all these chromosomes, known as the genome, resides within the nucleus of almost all cells of living eukaryotic organisms.

The spatial conformation of the genome is not random or ambiguous; rather, such conformation creates a specific 3D structure of chromosomes that serves specific purposes. Analogous to the significance attributed to the genetic information of DNA itself, the layout of the genome is also accredited certain significance [1]. 3D genome structures are an important aspect of study because they assist in the understanding of crucial topics regarding the genome, including: spatial gene regulation, transcription efficiency, genome interpretation, function implication, disease diagnosis and treatments, and drug design [2, 3].

The layout of genome structures depends on two important relationships: inter- and intra-chromosomal contacts, where a contact, also known as an interaction, refers to a spatial proximity (e.g. a short spatial distance below a threshold) between two regions on two different chromosomes or the same chromosome. Inter-chromosomal contacts refer to interactions amongst different chromosomes whereas intra-chromosomal contacts refer to interactions within one specific chromosome, or how each chromosome interacts with itself [1, 3]. Therefore, each individual chromosome has a 3D structure that, combined, form the 3D genome structure. Just like the entire genome, the 3D structure of each chromosome is also significant in the understanding of the important topics described [4].

Various techniques aimed at determining inter- and intra-chromosomal contact data, known as chromosomal conformation capturing techniques, which include 3C, 4C, 5C, and Hi-C, have seen significant progress in research and development in recent years. By using conformation capturing techniques to find intra-chromosomal contact data, it is possible to determine 3D structures of individual chromosomes. Therefore, the recent increase in opportunities to determine such chromosomal contact data has also increased the possibilities of determining the 3D structures of genomes and chromosomes [4]. One particular chromosomal conformation capturing technique applied here is Hi-C, which can determine both intra- and inter-chromosomal contact data at the genome wide scale [4].

Recently, a number of computational methods, such as the MCMC5C method based on 5C data [5], gradient descent based method [1], Integrated modeling platform also based on 5C data [6], and the Bayesian 3D Constructor (BACH) from Hi-C data [7], have been developed to reconstruct 3D models of a chromosomal region or the entire chromosome from intra-chromosomal contact data. Here, we develop a new computational methodology, called Gen3D, to utilize Hi-C data to produce 3D models of chromosome structures.

## Methods

### Overview of methodology

**Fig. 1:**
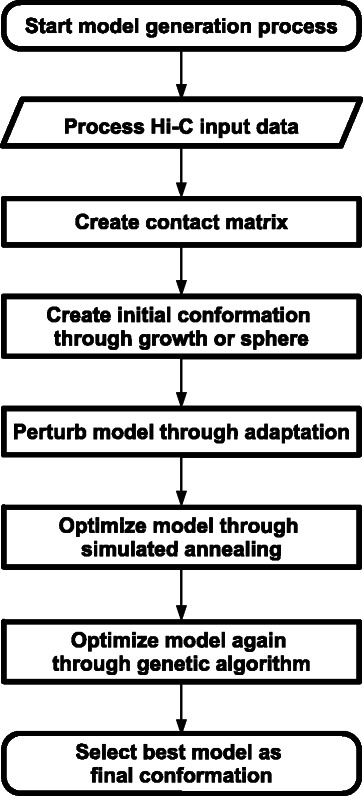
Flow chart of Gen3D methodology. Detailed flow chart illustrating the relationship between the steps and processes of the Gen3D methodology

Our approach to modeling 3D structures of chromosomes, called Gen3D, first involved processing Hi-C chromosomal contact data. As our input data, we used the aligned Hi-C data of a normal human B-cell, from the data of Lieberman et al. [4], and of a malignant human B-cell of an acute lymphoblastic leukemia patient, from the data of Wang et al. [3]. The processing of such Hi-C data resulted in a contact matrix. The contact matrix resembles an overview of all the contacts of a chromosome derived from the Hi-C input data. A 3D model of a chromosome can be created using the chromosome's contact matrix. But first an initial randomized structure of the chromosome must be created. Such initial structure was constructed using one of two strategies: a technique known as “growth” [8] or a randomized sphere. Once the initial model was created, three optimization algorithms were then applied sequentially to the random model to increase the model’s accuracy. The three optimization algorithms implemented were adaptation [8], simulated annealing [9], and genetic algorithm [10]. The final result was a sole 3D model of a chromosome structure. The flow chart shown below in Fig. 1 illustrates the modeling methodology and steps used in our approach. Furthermore, to quantify our results and to aid in the optimization techniques used, a scoring system representing the accuracy of the generated models was created. The following is a detailed description of the scoring system along with the chronological presentation of each main step of the various procedures and techniques used in the Gen3D methodology.

### Hi-C data processing

**Fig. 2:**
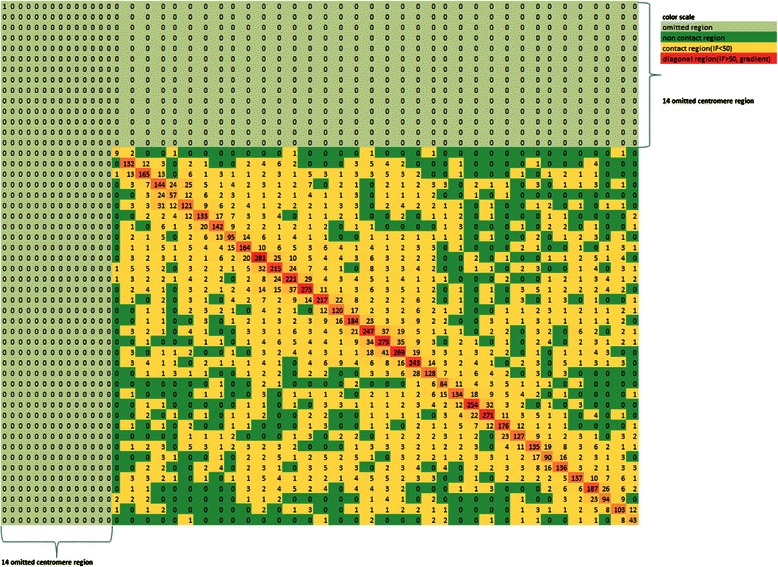
Contact matrix of chromosome 22. A contact, or interaction, matrix derived from Hi-C data of chromosome 22. The x and y-axes correspond to the regions of the chromosome. The number value of each element denotes the number of contacts for the corresponding regions. Here the yellow regions are contact regions: regions with at least one recorded contact; dark green regions are non-contact regions: regions with zero recorded contacts; light green regions are centromere regions: regions with no contact data; and orange regions are diagonal regions that correspond to the same regions of the chromosome and therefore aren’t considered

We began the 3D chromosome modeling procedure by processing the input Hi-C chromosomal contact data of the chromosome. The input data comprised a sequential list of all the contacts for a particular chromosome, where a contact refers to a spatial proximity (e.g. a short spatial distance below a threshold) between two regions on the physical structure of the chromosome. The list of contacts from the input data was gathered from the Hi-C conformation capturing technique [4]. The content of each contact from the input data contained three pieces of information: the chromosome of the specified contact and the two genomic locations corresponding to the two locations of the contact. From the entirety of the data, a contact (or interaction) matrix was formed for the chromosome. Each contact matrix resembles an overview of the contacts for that chromosome. Figure 2 shows an example of a contact matrix of chromosome 22.

The contact matrices were developed by first finding the number of loci of each chromosome. A locus, also known as a region of a fixed length (e.g. 1 Mb), is a small and specific part of the chromosome. The number of regions, or loci, per chromosome varies and is based on the size of the chromosome and the resolution (i.e. the fixed length of a region). The number of regions was obtained by subtracting the lowest genomic location found from the highest genomic location found in the list of genomic locations from the input Hi-C contact data. Then, that value (e.g., the length of the chromosome) was divided by the resolution to obtain the number of regions in the chromosome. The resolution in our case was 1 megabase (Mb). The contact matrix utilized the number of regions in the chromosome as the x and y-axes. For example, the contact matrix for chromosome 22 was found to contain 50 total regions of 1 Mb, and therefore had 50 elements for both the x-axis and y-axis. Figure 2 shows the contact matrix of chromosome 22 with the 50 elements for the x-axis and y-axis. The number of regions in other chromosomes ranged from 27 (chromosome 21) to 241 (chromosome 2).

The content of a contact matrix consisted of a count of the total number of contacts recorded (i.e. interaction frequency) for the corresponding regions from the input data. This count was calculated during the processing of the Hi-C data. First, for each contact processed of the input Hi-C data, the two regions of the contact were determined based on the two corresponding genomic locations. Then, the corresponding element in the contact matrix that correlated to the two regions of the contact was found. The value for this element was then increased by one for each contact recorded. Thus, the element in row i and column j of a contact matrix contained the quantity of contacts that were recorded in the input data for regions i and j. After processing the entirety of the Hi-C data, the contact matrix contained the total number of contacts for every combination of regions.

If an element of a contact matrix contained a number greater than zero, then that region was considered to be a 'contact region' because it contained at least one contact. Conversely, an element that contained the number zero was considered to be a 'non-contact region' because it contained no contacts. This is displayed in the example contact matrix of chromosome 22, shown in Fig. 2, in which contact regions are indicated by the yellow color and have a number value greater than zero, and non-contact regions are indicated by the dark green color and have a number value of zero. Diagonal regions, indicated by the orange color, represent contacts from the same regions and are therefore not relevant.

The input Hi-C chromosomal contact data contains some biases such as GC content, mapping, and sequence uniqueness. A normalization of the input data before utilization would sometime reduce these biases [11]. In this work, we applied two normalization techniques (Tuan et al. [1] and Imakaev et al. [12]) to normalize the Hi-C data and compared the models built from the normalized data with those from the initial un-normalized data.

There exists another problem when processing Hi-C data of chromosomes: the centromere regions of the chromosomes are not recorded. The centromere is the region of the chromosome where the two sister chromatids are bound together. As a result, no contact data was recorded for centromere regions and thus those regions required being omitted from the model as suggested by Tuan et al. [1]. After omitting the centromere regions, the total number of 1 Mb sized regions was reduced by the number of centromere regions. As a result, the contact matrices also shrunk according to the value of the corresponding chromosome's centromere regions. The example contact matrix of chromosome 22 shown in Fig. 2 has 14 omitted centromere regions represented by the light green regions on the top and on the left. Thus, Chromosome 22 has only 36 relevant regions after removing the 14 centromere regions.

The construction process of a chromosomal model, which is further described in subsequent sections, involved plotting each loci, or region, of the chromosome, on a 1 Mb scale, on a 3D coordinate system. The distance between each region was determined by that chromosome's contact matrix. The objective of our modeling process was to reconstruct 3D models of chromosomes, each represented as a collection of spatial positions of its loci that satisfy the intra-chromosomal contacts between loci as well as possible.

### Scoring functions

Once a model has been created, it is crucial to determine the effectiveness and efficiency of the model constructed by the employed methodology. This is performed by determining the level of congruency between the constructed model and the contact matrix derived from the Hi-C input data. This is represented using scoring functions. Scoring functions are needed to numerically assess the accuracy of the 3D models created by providing a quantitative representation of the success rate.

In addition, scoring functions are necessary for the function of the methodology. The optimization algorithms utilized (adaptation, simulated annealing, and genetic algorithms) all require scoring functions to be used as a conditional for structure improvement by determining whether the value of the score of the structure has increased. This in turn controls further actions of the algorithms. Therefore, we devised a scoring function to quantitatively represent the level of congruency between the constructed model and the contact matrix, as well as to help implement and improve our methodology.

The scoring function implemented had four main components: a contact satisfaction score, a non-contact satisfaction score, an interaction frequency score, and a maximum and minimum distance satisfaction score. The scoring function produced a final score of the model in the form of a percentage that was calculated using a weighted average of all four of the sub-scores. The subsequent section contains a description of the four sub-scores and the final score calculation.

#### Contact satisfaction score

The contact satisfaction (CS) sub-score represents the percentage of initial contacts that were satisfied in the final model. Determining whether contacts were satisfied in the final model involved finding the distance between each pair of adjacent points of the model for points that corresponded to a contact region. If that distance was below a predefined distance threshold, then that particular contact was considered satisfied. Therefore a distance threshold parameter was required to find and calculate the CS score, as suggested by Tuan et al. [1]. In this work, the value of the square of the distance threshold used was 7 micrometers^2^ (μm^2^). The CS score was calculated with the formula:

$$ \mathrm{C}\mathrm{S}=\left( total\; contact\; satisfaction\; count\ast 100\right)\;/\;\left( total\; contact\; count\right) $$

The *total contact count* was the total number of contact regions in the contact matrix; therefore the *total contact count* was calculated from the input Hi-C data. The *total contact satisfaction count* was the total number of those contact regions that were satisfied in the final model; therefore the *total contact satisfaction count* was calculated from the finished model. The two values were divided and multiplied by 100 to get the final result in the percentage form.

#### Non-contact satisfaction score

The non-contact satisfaction (NS) score represents the percentage of non-contacts that were satisfied in the model. The NS score was calculated in a similar manner to the CS score. Non-contacts in the final model were considered satisfied if the distance between each pair of adjacent points corresponding to a non-contact region was above the predefined distance threshold value. Therefore the distance threshold parameter was also required to find the NS score, of which the same value, 7 μm^2^, was used. The NS score was calculated with the formula:

$$ \mathrm{N}\mathrm{S}=\left( total\;non- contact\; satisfaction\; count\ast 100\right)\;/\;\left( total\;non- contact\; count\right) $$

Like the CS score, the *total non-contact count* was calculated from the contact matrix whereas the *total non-contact satisfaction count* was taken from the finished model.

#### Interaction Frequency satisfaction score

The Interaction Frequency satisfaction (IF) score represents the percentage of contacts that were satisfied in the model, similar to the CS score, but weighted by the actual interaction, or contact, frequency. The IF score was calculated with the following formula:

$$ \mathrm{IF}=\left(\sum\;\left({C}_{\mathrm{ij}}\ast I{F}_{\mathrm{ij}}\right) \ast 100\right)/\left( total\; IF\right) $$

*C*_ij_ was 1 if the contact at points i, j was satisfied, and 0 if not; *IF*_ij_ was the interaction frequency count, or number of contacts recorded, at points i, j; and *total IF* was the total interaction frequency, or total number of contacts, of the entire contact matrix taken from Hi-C data. The ∑ (*C*_ij_ * *IF*_ij_) statement determined the sum of all of the contacts of the model, which had a corresponding region that was satisfied in the final model. That value was divided by *total IF* and multiplied by 100 to get the IF score in the percentage form. The distance threshold parameter of 7 μm^2^ was again required.

#### Maximum and minimum distance satisfaction score

This score shows the percentage of regions of the final model that satisfied the maximum or minimum distance restraints. The predefined values used for the restraints were 20.25 μm^2^ for the square of the maximum distant restraint and .2 μm^2^ for the square of the minimum distant restraint as suggested by Tuan et al. [1]. The result was a maximum and minimum satisfaction score (MS) which was calculated with the below formula:

$$ \mathrm{M}\mathrm{S}=\left( max\; and\; min\; distance\; satisfied\; count\ast 100\right)/\left( number\; of\; regions\right) $$

The max and min distance satisfaction count was determined by calculating the number of regions of the final model that fell within the bounds of the maximum and minimum distance restraints. This value was then multiplied by 100 and divided by the total *number of regions* of the Hi-C data to get the MS satisfaction score in the percentage form.

#### Combination of scores

To get the final score representation of a model, the four sub-scores were merged. The combination of the scores involved taking the weighted average of all sub-scores such that the weight of each sub-score was different. For our purposes, the weight used for the CS score for all chromosomes was 2; the weight used for the NS score for all chromosomes was 2; the weight used for the IF score for all chromosomes was 3; and the weight used for the MS score for all chromosomes was 1. Weights for scores were assigned to give preference to more important aspects. For example, the IF score best represents the model’s score through consideration of frequency of contacts, and was therefore given a higher weight. Whole integers weights were chosen for simplicity as we found that deviations of decimal values had little to no effect on the final scores.

### Model construction

A discussion of the actual model construction follows. The construction process is illustrated by the flow chart in Fig. 1, which displays a visual explanation of how the following steps were integrated. The model construction began, succeeding the processing of the Hi-C data, with a random initial model creation through the growth step or with a random sphere followed by the optimization of the model through the adaptation step, the simulated annealing step, and the genetic algorithm step. Resulting from such steps, which are explained below, was the final model conformation.

#### Growth

Before the content of the contact matrices can be utilized, an initial structure must be randomly created to act as the basis of the model. To create such initial structure, we began by plotting each 1 Mb sized region using a probabilistic technique known as growth. The growth step was inspired by similar applications for protein as described by Vendruscolo et al. [8].

As implied by the name, the growth step involved adding a single region at a time resulting in step-by-step growth of the model. We utilized the following equation to add each point:

$$ {R}_{\mathrm{i}}={R}_{\mathrm{i}-1}+r $$

where i represented the index of the points; R represented a specific point, which made R_i_ the added point, and R_i-1_ the previous point from which the new points was extended; and r represented the vector, by which point R_i_ was extended from point R_i-1_, with a random length in between 0 and 1 μm and a random direction. Unlike protein conformation creation, there is no known information about the vector r or the relationship between two adjacent points for chromosomes making the initial prediction of the 3D structures randomized and in need of further data processing, which was done and is described below.

As the growth stage is designed for proteins, chromosome modeling is different; therefore it is sometimes advantageous to skip the growth stage for chromosomes with under 200 regions, or loci. Instead, it is advantageous to start with a random, spherical initial structure as suggested by Vendruscolo et al. [8]. In our case, only chromosomes 1, 2, and 3 had more than 200 regions and therefore the growth stage was applied. For the other chromosomes, the growth step was skipped, and the next step, adaptation, was applied by using random points on a unit sphere with a radius of 1 μm as the initial conformation. To better exemplify this decision, we tested on data using the reverse strategy. Specifically, we applied a random sphere as the initial conformation for chromosomes 1, 2, and 3 and growth for the remaining chromosomes. As a result, the average score was 7.77 % lower for the contact satisfaction scores and 12.59 % lower for the non-contact satisfaction scores.

#### Adaptation

With the initial conformation of the model built as described above, the first optimization algorithm, known as adaption, was implemented. Adaptation involved a refinement of the structure through randomized, local moves determined by whether such move increased the accuracy, or score, of the model, hence the name “adaptation” as the structure 'adapts' to achieve the best result.

**Fig. 3:**
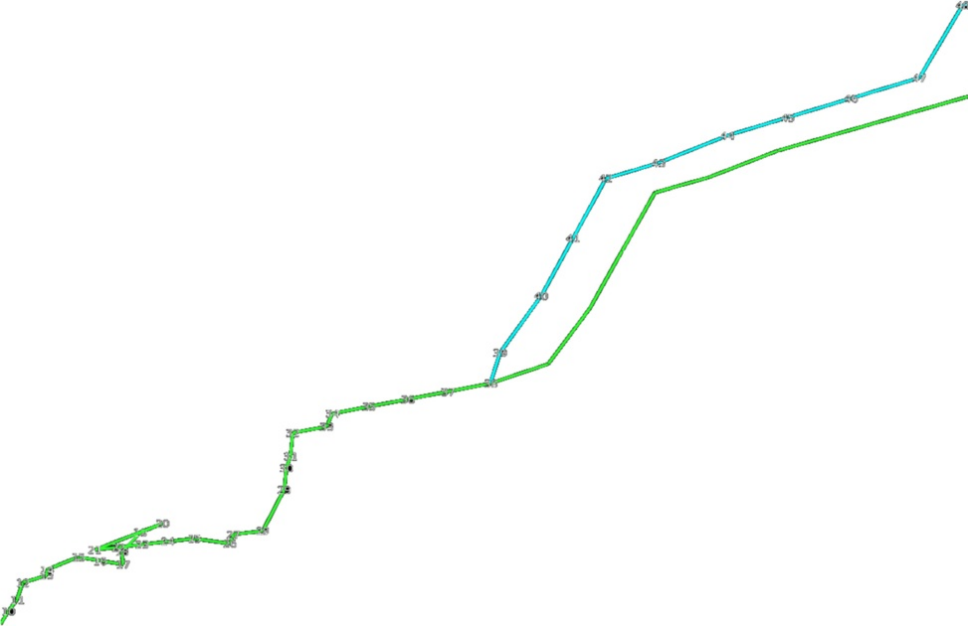
Visualization of the adaptation process. A visualization of a model undergoing the adaptation process. The model before undergoing adaptation is shown in light green and the model after resulting from the adaptation step is shown in light blue

To perform this step, we chose a random 1 Mb point, R_i_, along the structure and moved that point according to a random vector, r, which had the length of a random number between 0 and 1 μm and a random direction. Such movement was performed separately for 10 trials on the same point. The result was 10 possible movements for the point R_i_, and the best movement was chosen based on which increased the total score the most. Once the best movement was determined, the same translation, r, was then applied to the remaining points from R_i+1_ to R_n_, with R_n_ being the last point. Thus, the random vector r was added to R_i+1_, R_i+2_, R_i+3_, and so on up to R_n_. A visualization of the adaptation feature is represented in Fig. 3.

**Fig. 4:**
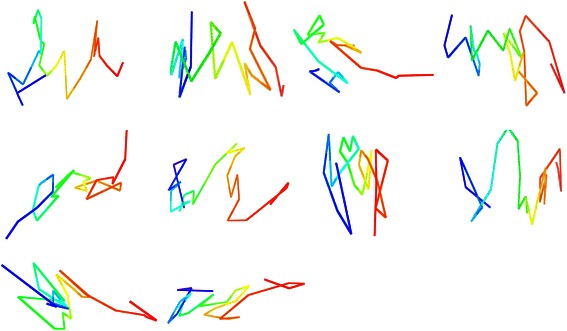
Visualization of successive iterations of adaptation step. Ten visualized models of chromosome 21 resembling different successive iterations of the adaptation step. Visualization is shown, from left to right and top to bottom, for every 5000 iterations to demonstrate the effect on the model the adaptation step has

This repeated movement on the entire model created one iteration of the adaptation step. To complete the adaptation stage, the process was repeated for 50,000 iterations. Every structure that resulted from a successful iteration of the adaptation step was saved separately. By the end of all the iterations, the result was an ensemble of different model structures, each having started from the same initial conformation structure but having completed a different variation of the adaptation step. Figure 4 shows ten visualized models of chromosome 21, the results of the adaptation stage after every 5000 iterations up to the 50,000^th^ iteration, to exemplify the progression.

#### Simulated annealing

After the adaptation step was completed, the next optimization procedure implemented was simulated annealing (SA) in accordance with Kirkpatrick et al. [9]. SA, inspired by the metallurgy procedure of similar name, involved searching for random new solutions, or new models, that were alternative to the current solution, or current model. If the new solution was better, in that it had a higher score, then the new solution was accepted as the current solution. However, if the new solution was worse, then such solution could still be accepted but with low probability. This action is the key feature of SA, differentiating it from similar, greedy optimization algorithms.

The purpose of accepting worse solutions, or less accurate models with a lower score, is to avoid 'local optimums' which are isolated extremes of efficiency. By doing such, it is possible to eventually arrive at an even better solution, or a more accurate model with a higher score, than the original as the SA process is performed multiple times, in our case 50 times. Not doing so, as is the case with the similar hill-climbing algorithm, could result in little to no progress from the original solution, as little or no immediate changes would result in a better solution.

To exemplify the benefits of SA as opposed to a hill-climbing algorithm, which is similar besides not accepting worse solutions, we can apply the procedure in analogy to the problem of climbing a mountain filled with numerous peaks in which the highest peak is the desired location. Every peak that isn’t the highest is a local optimum and a simple hill-climbing algorithm would stop at the first occurrence of a local optimum, as no immediate actions would result in immediate progress up the mountain. Rather, it is beneficial to descend from such peaks to eventually reach the highest peak, as is the case and benefit with SA.

A key feature of SA that makes this behavior possible is known as 'temperature' which comes from the origin of SA itself in which materials like glass or medals are repeatedly heated and cooled to change its properties, such process is known as annealing. In SA, the temperature, which is a numeric value, is set to a fixed value that gradually 'cooled', or decreased, over time. The value of this temperature helps determine whether or not the worse solution is accepted. This temperature value exists to set a soft limit on SA, as over time, the value of the temperature decreases more and more which causes the new solution to become less and less likely to be accepted. Additionally, the value of the difference between the current solution and new solution was also considered in making the decision to accept or not. A simple formula, which is a variation of the Boltzmann probability, utilized these factors to decide on whether to accept the worse solution:

$$ \exp \left(-\left(E\hbox{--} {E}_{\mathrm{i}}\right)\;/{T}_{\mathrm{i}}\right) $$

where E represented the score of the current solution; E_i_ represented the score of the potential new solution; and T_i_ represented the temperature when E_i_ was found. If this value was greater than a random number between 0 and 1, then the new solution was accepted as the current solution. The process was then repeated with the new solution.

With regards to SA being applied to 3D chromosome modeling, each model in the ensemble of models was independently compared to a new model that was generated using the adaptation procedure, described earlier. If the new model had a higher score then it was accepted as the current model, but if the new model had a lower score then it was accepted if it satisfied the Boltzmann probability equation. After 50 iterations of SA, the result was a collection of refined models each with a higher score than before.

#### Genetic algorithm

Following simulated annealing, the next optimization procedure implemented was genetic algorithm (GA) in accordance with Fonseca et al. [10], which was implemented to achieve better scores of 3D models. GA consists of three basic steps: natural selection, crossover, and mutation. These three processes prove to be beneficial optimization techniques due to their inspirations from evolution. The evolutionary process in nature, which involves the three processes of the same name, succeeds in yielding generations of species more advantageous than the previous. By mimicking the evolutionary process via computational means, the same results are achieved for 3D models because the general principles of evolution still apply in a computational setting. Following is a description of the three GA processes applied in our approach: natural selection, crossover, and mutation.

After previous optimization from SA, the result was a group of refined structures. The first step of GA, natural selection, was then implemented and involved reducing the produced ensemble to only the models with the highest of scores. The less accurate models, those with the lowest scores, were discarded as per the principle of the natural selection where only the most successful survive.

Once we were left with the most accurate models from the natural selection step, the next step in GA, known as crossover, was implemented. The crossover step involved swapping the coordinates of certain models at random crossover points. For example, consider models A_n_ and B_n_ both consisting of n points:

A_1_, A_2_, A_3_, … A_n-1_, A_n_

B_1_, B_2_, B_3_, … B_n-1_, B_n_

After implementing crossover with a crossover point at n = 2 the models would become:

A_1_, A_2_, B_3_, … B_n-1_, B_n_

B_1_, B_2_, A_3_, … A_n-1_, A_n_

The result of performing the crossover step on the models was an ensemble of 'children' models derived from the mixing of points from the 'parent' models. This is analogous to gene crossover during reproduction in nature.

The final step of GA, mutation, following natural selection and crossover, was lastly implemented. The mutation step involved tweaking the coordinates of one or more random points in each of the models. Tweaking of the point involved a random movement in a random direction for just that point, and the result was a slightly modified model. If such mutation increased the score of the model, then the mutation was kept. The mutation step was performed over ten thousand times with every model to achieve more accurate models.

GA was the last implemented optimization algorithm. GA followed SA because the one of the prime purposes of GA is to reduce an ensemble of models to only the most accurate of models through the natural selection step. Consequently, SA generates this ensemble of models therefore GA must follow SA to be most effective.

The implementation of GA turned a group of refined chromosome models into a more selective, integrated, and improved group that only contained the best models as per natural selection; contained models with swapped portions from cross over; and contained points that were further improved through mutation. The result from the completion of GA, and the entirety of implemented optimization procedures, was this improved collection of 3D chromosome models.

#### Final conformation

The result from the implementation of the optimization procedures was an ensemble of possible structures, having been improved and altered through the adaptation, simulated annealing, and genetic algorithm processes. To achieve a sole 3D model of the chromosome in consideration, the model with the highest score amongst the remaining models was selected as the final chromosome structure representation.

## Results and discussion

### Overview of results

To execute our methodology described we used the Hi-C data of a normal human B-cell, from the data of Lieberman et al. [4], as well as the Hi-C data of a malignant human B-cell of an acute lymphoblastic leukemia patient, from the data of Wang et al. [3], as the input data. The output was 3D models of 23 chromosome structures from both sets of input data. We utilized the scoring system afore mentioned to quantify the results of our models to get a better representation of the accuracy. We also employed the use of heat maps to create matrix-based representations of the accuracy of the models. Finally, we visualized the 3D chromosomal models themselves to get a visual representation of the model and to compare with other methods. The results from our experiment are presented via the scoring system, heat maps, and visualizations below, along with various algorithm validation techniques such as convergence testing and robustness by recovery testing. Various made comparisons with similar experiments, which serve the purpose of validating and placing our approach, are also presented below. Finally, some inconsistencies and areas of future study are discussed.

### Algorithm validating

**Fig. 5:**
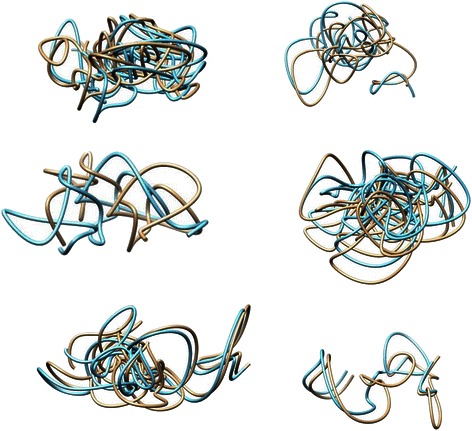
Convergence testing results. Six pairs of two separate models generated side by side for convergence testing comparison. From left to right, top to bottom, the chromosomes used are chromosome 16, 19, and 21 from the cancerous cell data, followed by chromosome 14, 15, and 21 from the normal cell data

To validate and test the success and consistency of our algorithm, we performed convergence testing which involved generating multiple models of the same chromosome then comparing the various versions of the results. Further evidence for the consistency and success of our algorithm would be provided if the separately generated models were similar in terms of score and visualization. The juxtaposition of the visualization for two trials for various chromosomes is shown in Fig. 5. From left to right, top to bottom, the chromosomes used for convergence testing are chromosome 16, 19, and 21 of the cancerous cell [3], followed by chromosomes 14, 15, and 21 of the normal cell [4]. As is evident through the figure, each trial of convergence testing resulted in the two models having similar 3D structures. For visualization, the UCSF Chimera [13] tool was used. The similarity between each of the pairs of models derived separately from the same chromosome contact data shows the consistency and success of the methodology.

**Fig. 6:**
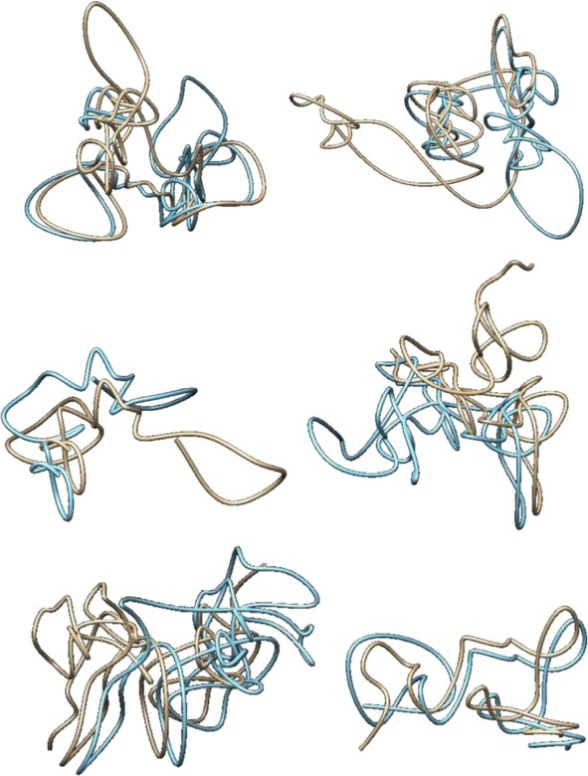
Robustness by recovery testing results. Six pairs of two separate models generated side by side for robustness by recovery comparison. From left to right, top to bottom, the chromosomes used are chromosome 19, 20, and 21 from the normal cell data, followed by chromosome 19, 20, and 21 from the normal cell data

To test the robustness of our algorithm, recovering capability was checked as explained by Baù et al. [6]. Recovering capability first involved generating a model using Hi-C data. After that, a contact matrix was created from that generated model using contact and non-contact satisfaction data. Using that contact matrix another model was generated using our methodology. The visualization of the two models, before and after, were then compared. Several examples of recovering capability are shown in Fig. 6. From left to right and top to bottom, the shown models are of chromosome 19, 20, and 21 of the normal cell [4], followed by chromosome 19, 20, and 21 of the cancerous cell [3]. For visualization, the UCSF Chimera [13] tool was used. From the figure, it is apparent that the two generated models for each chromosome are quite similar in structure. The similarity between two models of the same chromosome derived before and after a contact matrix shows the robustness of the methodology.

### Evaluation of scores of models

As described before, a scoring system was created to numerically assess the accuracy of the 3D models created and provide a quantitative representation of the success rate. Table 1 shows the score representations of the results from our input data, the malignant B-cell from the data of Wang et al. [3], by showing the CS score, NS score, and IF score of the resulting models. The value of the square of the distance threshold parameter was 7 μm^2^ as suggested by Tuan et al. [1], and was used to find these scores. The average score for the CS score across all chromosomes was 77.68 and 76.23 % for the NS score. The average score for the IF score was 92.05 %. Table 2 shows the same score representations only using the normal B-cell input data from the published Hi-C data of Lieberman et al. [4]. Additionally, the average distance of unsatisfied contacts and the average distance of unsatisfied non-contacts are also recorded in this table. Here the average scores for the CS score for all chromosomes was 80.41 and 80.81 % for the NS score. And the average score for the IF score was 79.41 %. The average of the average distance of unsatisfied contacts across all chromosomes was 11.30 μm and, alternatively, 4.16 μm for non-contacts. The resulting high percentages for the CS, NS, and IF scores demonstrate the effectiveness of our methodology.

**Table 1:** Scores of models from malignant B-cell Hi-C data of Wang et al. [3]

Chromosome	Contact satisfied score (%)	Non-contact satisfied score (%)	IF satisfied score (%)
1	71	70	89
2	66	66	87
3	69	71	88
4	73	74	91
5	70	69	90
6	73	73	91
7	72	70	91
8	70	80	89
9	84	70	96
10	72	70	91
11	76	73	93
12	72	72	90
13	81	76	94
14	81	79	95
15	73	73	92
16	80	78	96
17	74	73	92
18	93	90	97
19	82	74	90
20	90	82	92
21	95	99	96
22	92	95	95
Average	77.68	76.22	92.05

Column 1: list of chromosomes in chronological order; Columns 2: the percentage of satisfied contacts; Columns 3: the percentage of satisfied non-contacts; Columns 4: the percentage of satisfied interaction frequencies; all for models of 22 pairs of chromosomes from the Hi-C data of Wang et al. [3]. Average of scores is also recorded in the last row

**Table 2:** Scores of models from normal B-cell Hi-C data of Lieberman et al. [4]

Chromosome	Contact satisfied score (%)	Non-contact satisfied score (%)	IF satisfied score (%)	Avg. of Dist. of unsatisfied contacts (μm)	Avg. of Dist. of unsatisfied non-contacts (μm)
1	79	79	77	11.26	3.95
2	78	78	77	12.36	3.89
3	75	75	78	11.95	3.81
4	83	84	81	10.29	4.49
5	82	85	80	10.98	4.27
6	79	79	80	10.88	4.33
7	79	79	78	12.12	3.54
8	82	85	80	10.47	4.34
9	82	86	80	12.17	4.05
10	79	80	78	11.61	3.97
11	77	77	77	11.52	3.75
12	79	78	80	12.41	4.22
13	80	81	79	10.28	4.43
14	75	75	78	13.21	4.31
15	79	81	78	11.48	4.49
16	83	81	81	10.07	4.08
17	80	79	79	10.11	4.73
18	77	78	78	11.4	3.24
19	83	86	80	11.44	4.34
20	78	78	79	11.97	3.5
21	91	89	85	11.28	5.05
22	89	85	84	9.38	4.69
Average	80.41	80.82	79.41	11.30	4.16

Same legend as Table 1 only using 22 chromosomes from the Hi-C data of Lieberman et al. [4] and including additional columns showing the average distance of unsatisfied contacts and the average distance of unsatisfied non-contacts

Normalization in accordance with Imakaev et al. [12] was implemented in hopes of achieving better scores through eliminating certain problems associated with bias and noise such as GC content, mapping, and sequence uniqueness [11]. The resulting scores from normalized data were evaluated to determine the effectiveness of normalization. From the normalized cancerous cell data of Wang et al. [3] the average CS score for all chromosomes was 79.64 %, the average NS score was 42.04 %, and the average IF score was 84.32 %. The results for all chromosomes of the normalized data of the cancerous cell are presented in Table 3. The non-normalized results from the same dataset were 77.68 % for the CS score, 76.22 % for the NS score, and 92.05 % for the IF score. Although the CS score was higher for the normalized data, the NS and IF scores were lower, therefore the applied normalization technique did not improve the overall scores of the cancerous cell data. From the normalized normal cell data of Lieberman et al. [4] the average CS score for all chromosomes was 80.5 %, the average NS score was 50.04 %, and the average IF score was 86.04 %. Table 4 shows the results of the normalized data from the normal cell for all chromosomes. The non-normalized results were 80.41 % for the CS score, 80.82 % for the NS score, and 79.41 % for the IF score. The average scores actually improved slightly, by 0.09 %, for the CS score and decently for IF score, by 6.63 %. However the NS score dropped considerably, by 30.78 %, therefore the overall score was lower for the normalized data. Overall, the outcome of applying normalization to our method does not consistently increase the overall scores of 3D chromosome models. However, this observation may be specifically related to our method as it has been shown that the normalization of Hi-C data can improve 3D genome models with other methods [1].

**Table 3:** Scores of models from normalized Hi-C data of Wang et al. [3]

Chromosome	Contact satisfied score (%)	Non-contact satisfied score (%)	IF satisfied score (%)
1	81	50	91
2	75	41	85
3	72	41	76
4	76	41	100
5	78	41	85
6	71	41	89
7	71	41	85
8	94	42	92
9	93	40	90
10	73	40	86
11	69	42	86
12	80	41	100
13	88	40	88
14	95	42	96
15	80	41	90
16	66	42	76
17	91	41	88
18	60	41	69
19	67	42	23
20	93	40	85
21	96	50	100
22	83	45	75
Average	79.64	42.04	84.32

Same legend as Table 1 only with the normalized Hi-C data from Wang et al. [3]

**Table 4:** Scores of models from normalized Hi-C data of Lieberman et al. [4]

Chromosome	Contact satisfied score (%)	Non-contact satisfied score (%)	IF satisfied score (%)
1	66	40	78
2	60	47	73
3	69	40	76
4	78	41	100
5	82	41	93
6	65	48	83
7	73	46	83
8	91	41	92
9	91	40	94
10	73	57	87
11	67	44	80
12	80	41	100
13	80	48	88
14	96	75	95
15	80	50	89
16	86	45	91
17	80	44	29
18	93	100	90
19	87	60	92
20	89	57	92
21	89	46	92
22	96	50	96
Average	80.5	50.04	86.04

Same legend as Table 2 only with the normalized Hi-C data from Lieberman et al. [4]

### Heat map representation

**Fig. 7:**
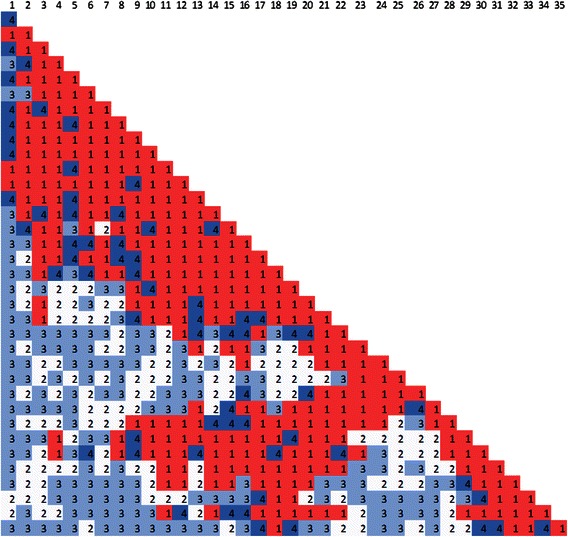
Heat map representation for chromosome 22. Heat map representation of model of chromosome 22 where 1 (red) represents a contact satisfied, 3 (light blue) represents a non-contact satisfied, 2 (white) represents a contact unsatisfied, and 4 (dark blue) represents a non-contact unsatisfied. X and y-axes resemble each region of the chromosome

To further demonstrate the accuracy of our generated models, we created a heat map representation of the satisfied and unsatisfied contact and non-contact regions for each model. Heat map representations are effective at representing the accuracy of the model. The heat map shows a matrix in which the regions of the resembled chromosomes are displayed as the x and y-axis, similar to the contact matrix. The content of the elements of the heat map resembled a 1 (background of red) if the corresponding regions indicated a contact satisfied; a 3 (light blue) if the corresponding regions indicated a non-contact satisfied; a 2 (white) if the corresponding regions indicated a contact unsatisfied; and a 4 (dark blue) if the corresponding regions indicated a non-contact unsatisfied. Figure 7 shows a heat map representation of the model generated for chromosome 22. Figure 7 visually demonstrates the large percentage of satisfied contact and non-contact regions through the red and light blue regions.

### Visualization of models

**Fig. 8:**
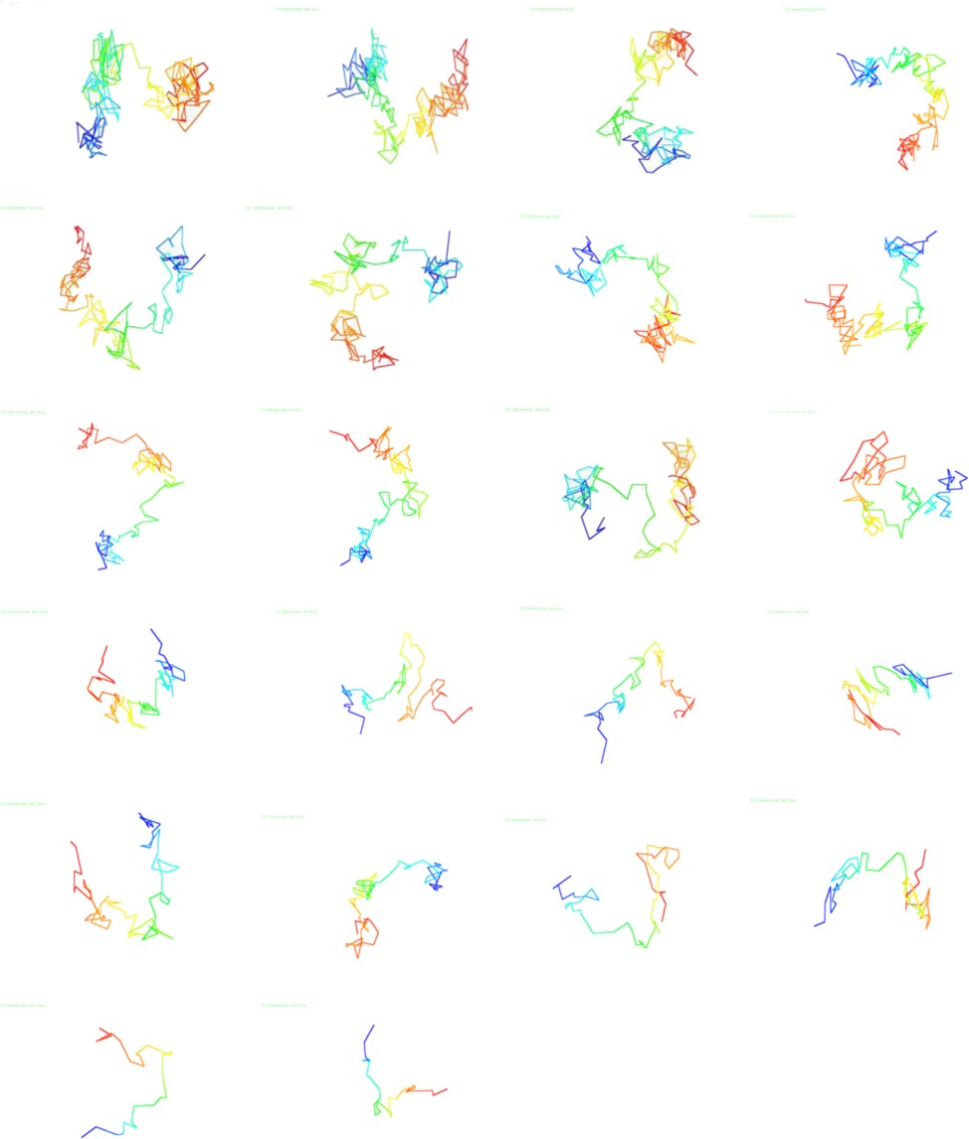
Visualized chromosome models of Hi-C data from Wang et al. [3]. Chronological order from left to right and top to bottom, sequentially, of visualized models of the 22 chromosomes from the Hi-C data of Wang et al. [3]

**Fig. 9:**
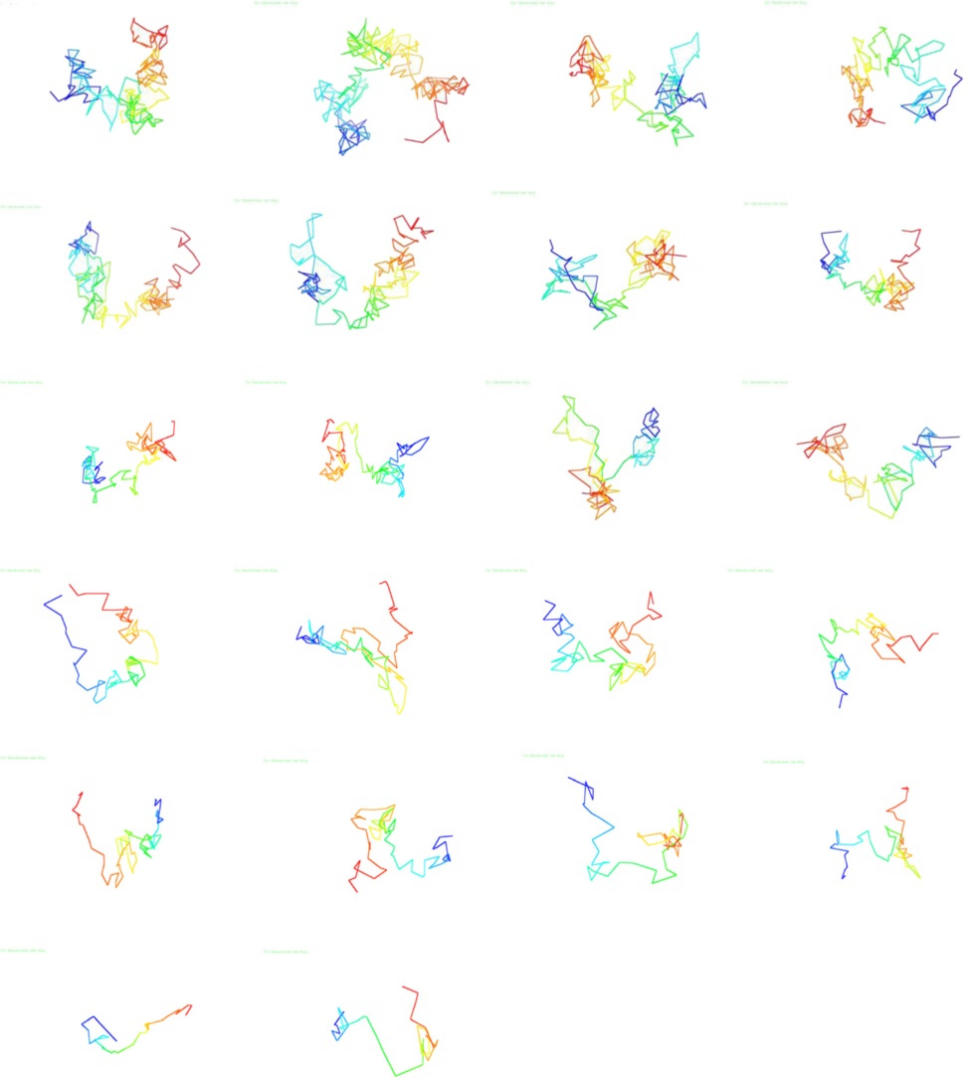
Visualized chromosome models of Hi-C data from Lieberman et al. [4]. Chronological order from left to right and top to bottom, sequentially, of visualized models of the 22 chromosomes from the Hi-C data of Lieberman et al. [4]

To get an idea of what the generated 3D models looked like, we visualized the models using the 3D coordinates from the output data of our methodology. Visualizing the generated 3D models is also important for performing comparisons with different 3D chromosome model generation methods. Using the Pymol tool [14] for visualization, chromosomes 1 through 22 from both data sets were visualized and are shown in Figs. 8 and 9. The models of Fig. 8 were generated from the published Hi-C data of Wang et al. [3] and the models of Fig. 9 were generated from the published Hi-C data of Lieberman et al. [4]. Additionally the PDB files containing 3D coordinates of all models used for visualization are published on our sourceforge site.

### Comparison with MCMC5C

**Fig. 10:**
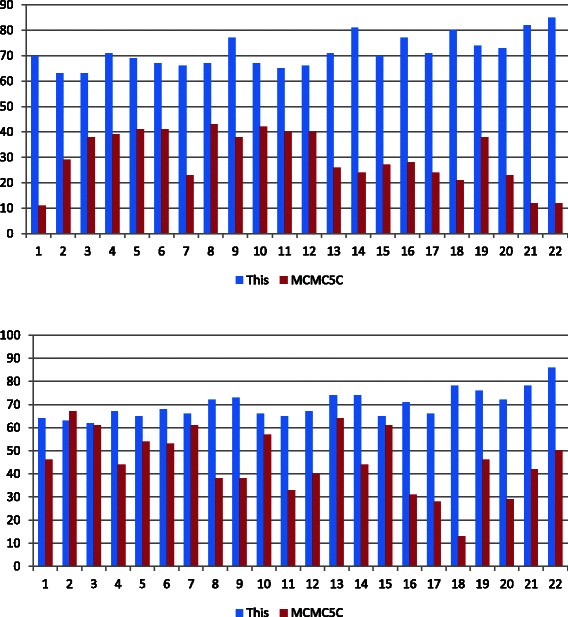
Comparison with MCMC5C. Bar graphs representing comparison of scores for the MCMC5C approach [5] and our approach, Gen3D. Bar graph on top shows comparison of CS score, and bar graph on bottom shows comparison of NS score. Score percentage is represented in the y-axis and the chromosome number is represented in the x-axis

**Fig. 11:**
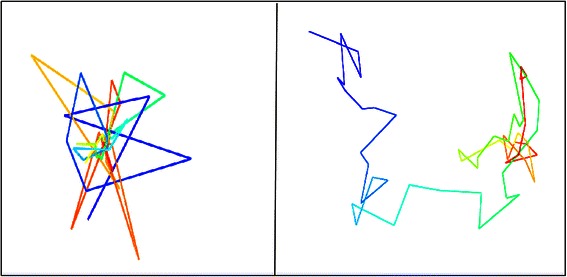
Comparison with MCMC5C of chromosome 19. Side by side comparison of visualized models of chromosome 19. The model on the left is from the MCMC5C method [5] and the model on the right is from our Gen3D method

To better understand the effectiveness of our approach, we compared our results with the results from a similar approach known as Markov chain Monte Carlo 5C (MCMC5C) that was also designed to create 3D chromosome structures [5]. Using the MCMC5C approach with the code and data published by Rousseau et al. [5], combined with our Hi-C data and appropriate parameters, we generated 3D models of all chromosomes. Using 100 million iterations an ensemble of 100 structures was generated, of which the scores were calculated for the best structures. The juxtaposition of the CS and NS scores of the generated models for both the MCMC5C method and our method is shown in the form of bar graphs in Fig. 10. As is clear by the graphs, our approach provided consistently better scores that are significantly higher for most chromosomes, including a better contact score for every chromosome and a better non-contact score for every chromosome except for chromosome 2. The contrast is also represented through the comparison of the visualized model of chromosome 12 in Fig. 11. Here the MCMC5C method is represented on the left and our Gen3D method represented on the right. It is apparent from the visualized models that the two approaches produced models with obvious differences. Here, the Pymol tool [14] was used for visualization of the models.

### Future studies

A number of additional future areas of study arise from this experiment. One such possible direction to investigate in the future is the incorporation of additional optimization techniques into the methodology that would further enhance the score of the models and, therefore, the models themselves. A number of such techniques deserve attention to determine whether incorporation would be beneficial. For example, an optimization procedure known as conjugate gradient descent could prove beneficial and requires further investigation. Three additional possible areas of future study, that are described below, are the inconsistency of long range contacts, the application of 5C data and the application of high-resolution models.

### Long range contacts

With the results of implementing our methodology presented, a discussion of a minor inconsistency and limitation regarding our experiment is needed. Of which regards the difference between long range and short range interactions. As can be shown in the heat map representation of the contact satisfactions in Fig. 7, a larger amount of short range contact interactions are satisfied than the amount of long range contact interactions that are satisfied. Here short range interactions are contacts between regions relatively close together, defined by |i-j| < = 10 where i and j are the row and column index respectively of the heat map; and long range interactions are contacts between regions relatively far apart, defined by |i-j| > 10. The exact nature of this phenomenon requires further investigation to determine the cause and also to figure out possible solutions that can bridge the gap between the number of short range contacts satisfied compared to the number of long range contacts satisfied.

### Application of 5C data

Another possible area of future study involves utilizing additional chromosome conformation capturing techniques. Analogous to the Hi-C technique used to obtain chromosomal contact data, another technique known as carbon-copy chromosome conformation capture (5C) has a similar function. This was tested by Baù et al. [6] when they used such data to determine 3D structures of chromosomes, specifically chromosome 16. We tested the applicability of our methodology with 5C data rather than Hi-C data by generating a model of chromosome 16 with the published 5C data and compared the results with the generated model of Baù et al. [6]. The side-by-side comparison of the visualized results, with our model generated using the UCSF Chimera tool [13] show that the two models are similar**.** However, additional testing is required to ensure our methodology could work equally well with 5C data. In addition, the testing of the applicability of other chromosome conformation capturing techniques, such as 4C, would be beneficial and provide insight into the versatility of our methodology.

### High-resolution models

**Fig. 12:**
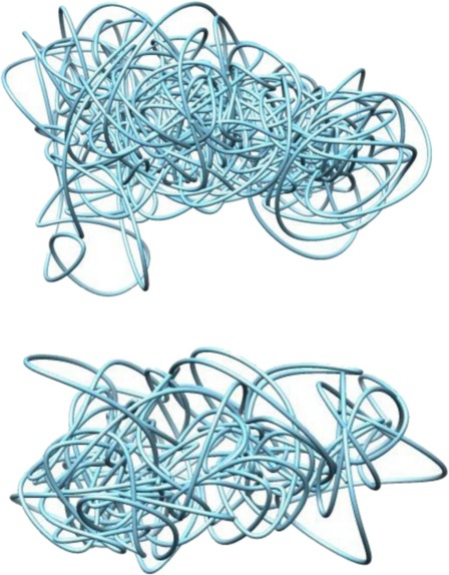
Visualization of chromosome 21 in higher resolutions. Visualization of two generated models of chromosome 21 from the cancerous cell data of Wang et al. [3]. Generated models are of higher resolution than originally tested. Model on top has a resolution of 100 K and model on bottom has a resolution of 200 K

One last area of future study involves generating chromosome models with higher resolutions. We have performed some initial analysis regarding constructing models with higher resolutions, however more investigation is required. Our analysis consisted of generating models of multiple chromosomes with resolutions of 100 K and 200 K, then comparing the results to the results from the standard resolution of 1 MB. The results of such comparison revealed that using higher resolutions yields less accurate results and larger running time required to build the models. We suspect this is due to the following reasons. First, since a higher resolutions means more units per model, the number of iterations for all steps might be too low, especially adaptation. We suspect additional iterations will yield higher scores. Second, the growth technique fails for models with higher resolutions due to the number of units; therefore spherical initial structure was used. This resulted in less accurate models as Vendruscolo et al. [8] suggests using growth for models with more than 200 units, which is not the case here. The visualization of our analysis is presented in Fig. 12 which shows two visualized models of chromosome 21 from the cancerous cell input data. The generated model on top has a resolution of 100 K and the model on bottom has a resolution of 200 K. Since our initial investigation demonstrated that models with higher resolutions resulted in lower scores and took more time, further investigating is needed to help improve the applicability of high-resolution models with our methodology.

## Conclusions

Achieving accurate models of chromosomal structures from intra-chromosomal contact data (interaction within each chromosome) helps in determining the nature of the structure of the entire genome, being the full set of chromosomes. Knowing the nature of genome structures has been proven to be a vital gateway in further understanding of the nature of the entire genome, representing benefits akin to knowledge of the genetic information itself. In particular, such information can lead to further information regarding genome processes and functions including spatial gene regulation, transcription efficiency, genome interpretation, function implication, disease diagnosis and treatments, and drug design, all of which have potentially crucial implications in the corresponding field. For example, information of genome structures of disease-prone organisms, such as viruses, can aid in diagnosis and treatment of such diseases. These benefits have created a need for accurate models of human chromosomes. Our computational approach helps to address this need. In addition, as 3D chromosome models can provide practicality and value to the research community by contributing to research efforts pertaining to genome functions and characteristics such as the key areas previously listed. Therefore, our approach can directly aid the research community as a reliable and efficient tool to achieve much needed accurate chromosome models.

To conclude, our approach, known as Gen3D, has been demonstrated to be an effective process for constructing 3D models of chromosomal structures. Therefore, we believe that our computational methodology will successfully provide a much-needed tool capable of building 3D models of chromosomal structures, which will provide insight into knowledge regarding human genomes and chromosomes as well as shed some light on further areas of study.

## References

[1] Trieu T, Cheng J. Large-scale reconstruction of 3D structures of human chromosomes from chromosomal contact data**.** Nucleic Acids Research. 2014. doi:10.1093/nar/gkt1411. 10.1093/nar/gkt1411 PMC3985632 24465004

[2] Taylor KH, Briley A, Wang Z, Cheng J, Shi H, Caldwell CW (2013). Aberrant Epigenetic Gene Regulation in Lymphoid Malignancies. Semin Hematol.

[3] Wang Z, Cao R, Taylor K, Briley A, Caldwell C, Cheng J (2013). The Properties of Genome Conformation and Spatial Gene Interaction and Regulation Networks of Normal and Malignant Human Cell Types. PLoS One.

[4] Lieberman-Aiden E, Van Berkum NL, Williams L, Imakaev M, Ragoczy T (2009). Comprehensive Mapping of Long-Range Interactions Reveals Folding Principles of the Human Genome. Science.

[5] Rousseau M, Fraser J, Ferraiuolo MA, Dostie J, Blanchette M (2011). Three-dimensional modeling of chromatin structure from interaction frequency data using Markov chain Monte Carlo sampling. BMC Bioinformatics.

[6] Baù D, Sanyal A, Bryan R, Lajoie EC, Byron M, Jeanne B, Lawrence JD, Marc A (2010). The three-dimensional folding of the α-globin gene domain reveals formation of chromatin globule. Nature.

[7] Hu M, Deng K, Qin Z, Dixon J, Selvaraj S, Fang J, Ren B, Liu JS (2013). Bayesian Inference of Spatial Organizations of Chromosomes. PLoS Comput Biol.

[8] Vendruscolo M, Kussell E, Domany E (1997). Recovery of protein structure from contact maps. Fold Des.

[9] Kirkpatrick S, Gelatt CD, Vecchi MP (1983). Optimization by simulated annealing. Science.

[10] Fonseca CM, Fleming PJ (1993). Genetic Algorithms for Multiobjective Optimization: Formulation, Discussion and Generalization. ICGA.

[11] Yaffe E, Tanay A (2011). Probabilistic modeling of Hi-C contact maps eliminates systematic biases to characterize global chromosomal architecture. Nat Genet.

[12] Imakaev M, Fudenburg G, McCord R, Naumova N, Goloborodko A, Lajoie B, Dekker J, Mirny L (2012). Iterative correction of Hi-C Data reveals hallmarks of chromosome organization. Nat Methods.

[13] Pettersen EF, Goddard TD, Huang CC, Couch GS, Greenblatt DM, Meng EC, Ferrin TE (2004). UCSF Chimera - A Visualization System for Exploratory Research and Analysis. J Comput Chem.

[14] Schrodinger LLC (2010). The PyMol Molecular Graphics System, Version 1.3.

